# Impact of Quiet Quitting and Nursing Work Environment on Quality of Care and Patient Safety: A Cross-Sectional Study Among Nurses in Greece

**DOI:** 10.3390/healthcare14142119

**Published:** 2026-07-15

**Authors:** Ioannis Moisoglou, Aglaia Katsiroumpa, Olympia Konstantakopoulou, Ioanna V. Papathanasiou, Evangelos C. Fradelos, Aris Yfantis, Panagiota Peleka, Ioanna Prasini, Anastasia Tsalouka, Petros Galanis

**Affiliations:** 1 Department of Nursing, University of Thessaly, 41500 Larissa, Greece; iopapathanasiou@uth.gr (I.V.P.); efradelos@uth.gr (E.C.F.); 2 Clinical Epidemiology Laboratory, Faculty of Nursing, National and Kapodistrian University of Athens, 11527 Athens, Greece; aglaiakat@nurs.uoa.gr (A.K.); olykonstant@nurs.uoa.gr (O.K.); gpeleka@nurs.uoa.gr (P.P.); anastasiatsalouka@yahoo.gr (A.T.); pegalan@nurs.uoa.gr (P.G.); 3 Directorate of Secondary Education of Fthiotis, Special Vocational Education Laboratory of Fthiotis, 35100 Lamia, Greece; arisyfantis@gmail.com; 4 Palliative Care Unit Galilee, 19004 Spata, Greece; iprasini@galilee.gr

**Keywords:** nurses, patients, quality of care, quiet quitting, safety, work environment

## Abstract

**Background/Objectives**: The quality and safety of care provision have consistently remained central concerns for the management of healthcare organizations worldwide. Nurses’ work environment and occupational behaviors may significantly affect these outcomes. The present study aimed to evaluate the nursing work environment, assess the extent of quiet quitting among nurses, and investigate their impact on the quality and safety of care delivery. **Methods**: A cross-sectional study was conducted in Greece, and data were collected via an online survey between October and December 2025. Quiet quitting and work environment were measured using the nine-item Quiet Quitting Scale (QQS) and the five-item short form of the Practice Environment Scale of the Nursing Work Index (PES-5), respectively. Multivariable logistic regression analyses were performed using IBM SPSS Statistics 28.0. The level of statistical significance was set at 0.05. **Results**: The sample included 492 nurses. The overall quiet quitting rating was low to moderate (mean = 2.18, SD = 0.65), and the mean score for the PES-5 was 2.44 (SD = 0.53) indicating a moderate realization of a supportive work environment. More than half of the participants (52.0%, n = 256) rated the quality of care at their unit as good, and 23.6% (n = 116) as fair. Additionally, 33.1% (n = 163) of the nurses described patient safety as good and 28.5% (n = 140) very good. In the multivariable model, lower scores for quiet quitting were significantly associated with higher likelihood of reporting perceived quality of care as good or excellent (adjusted OR = 0.429; 95% CI: 0.310–0.593; *p* < 0.001) and increased odds of reporting perceived patient safety as good or excellent (adjusted OR = 0.394; 95% CI: 0.281–0.553; *p* < 0.001). Higher PES-5 scores were linked to more than nine-fold increased odds of reporting good or excellent perceived quality of care (adjusted OR = 9.155; 95% CI: 5.374–15.596; *p* < 0.001) and an over twenty-fold increase in the odds of reporting good or excellent perceived patient safety (adjusted OR = 22.190; 95% CI: 11.533–42.693; *p* < 0.001). **Conclusions**: This study highlighted that low levels of quiet quitting and a more favorable nursing practice environment are important and independent predictors of better perceived quality and safety of care.

## 1. Introduction

The end of the COVID-19 pandemic marked a critical turning point for the nursing profession. Nurses, already working in highly demanding environments, faced exceptionally challenging conditions throughout the pandemic and experienced the cumulative effects of these longstanding and pandemic-related pressures [1]. Burnout, mental health challenges, and leaving the profession have emerged as the predominant issues affecting the nursing workforce [2,3,4], with the organization and functioning of their work environment being associated with the aforementioned conditions [5]. During the COVID-19 pandemic in the United States, approximately 100,000 nurses resigned from the nursing profession, while nearly ten times more are projected to leave by 2027. This phenomenon is widely referred to as the Great Resignation [6]. Many nurses who remain in the profession, however, may opt for quiet quitting by reducing their work performance in an effort to protect themselves from demanding working conditions and their adverse consequences [7]. This choice, however, may compromise the quality and safety of patient care. Alongside nurses’ work-related behaviors, organizational factors within the nursing practice environment may also significantly influence care quality and patient safety.

Quiet quitting entered the organizational discourse during the period of disruption caused by the COVID-19 pandemic, as employees increasingly reconsidered the boundaries between professional demands and personal well-being. Early empirical work focused mainly on corporate and business settings, where the phenomenon was first systematically described and measured [8]. More recent findings, however, suggest that quiet quitting is also highly relevant to healthcare. Nurses appear to be particularly affected, with studies reporting that a substantial proportion of them display quiet quitting behaviors, often at rates higher than those observed among other healthcare professionals [7,9,10]. Quiet quitting reflects a state of reduced work engagement, in which employees remain formally present in their roles but psychologically and behaviorally distance themselves from their work [11]. Rather than expressing engagement, initiative, or willingness to contribute beyond basic expectations, individuals who quiet quit restrict their effort to what is strictly necessary for role compliance [8,12]. In practical terms, this may be expressed through avoidance of discretionary tasks, reluctance to accept extra duties or shifts, limited participation in activities outside the core job description, and unwillingness to provide additional work input, even when workplace demands temporarily increase [11].

Nursing is a highly demanding profession that extends well beyond the routine provision of care. It requires continuous patient monitoring and sustained vigilance to prevent complications and adverse events, such as falls and infections, and to ensure timely interventions that mitigate their consequences. Therefore, although quiet quitting may represent an effort by nurses to achieve a balance between the demands of their professional and personal lives, such an attitude may nevertheless jeopardize patient safety and undermine the quality of care. A leadership-supportive work environment characterized by interprofessional collaboration, recognition of nurses’ role, and reduced workload may decrease nurses’ intention to engage in quiet quitting [13,14,15].

The nursing work environment has long been a focus of research. Although early studies primarily examined its influence on nurses’ intention to leave their employing organizations [16], subsequent evidence has demonstrated its substantial impact on patient outcomes [16]. A supportive nursing work environment is strongly dependent on the availability, development, and effective integration of nursing personnel. Adequate staffing is particularly important, as patients cared for in understaffed units are more likely to experience adverse outcomes, including higher mortality, hospital readmission, and longer length of stay [17]. Beyond staffing levels, the organizational recognition of nurses’ expertise is also essential. Involving nurses in hospital committees and decision-making structures may strengthen the assessment and continuous improvement of care quality and patient safety [18]. In daily clinical practice, nurses also need consistent managerial support, guidance in managing stressful and complex working conditions, and access to opportunities for professional learning, career progression, and innovation. Such conditions can promote both professional development and better service delivery. Leadership that creates an empowering and supportive practice environment may improve nurses’ job satisfaction and structural empowerment, thereby contributing to safer and higher-quality care [19,20]. Interprofessional collaboration is another key element of the nursing work environment. Given that nurses and physicians are primary providers of direct patient care, effective communication, mutual respect, and teamwork between these professional groups are critical for promoting patient safety [21,22].

The nursing work environment represents a complex and multidimensional construct, whereby each of its individual characteristics, and ideally their collective presence, shapes a healthy workplace. This, in turn, is closely linked to two principal outcomes: the safeguarding of nurses’ occupational well-being and the advancement of care quality and patient safety [23]. Within this context, the present study aimed to evaluate the nursing work environment, assess the extent of quiet quitting among nurses, and investigate their impact on the quality and safety of care delivery in a sample of nurses in Greece. As we mentioned above, the association between work environment and quality and safety of healthcare is well-established. Although the relationship between the nursing work environment and care outcomes has been widely investigated, empirical evidence on the potential impact of quiet quitting on perceived healthcare quality and patient safety remains limited. Therefore, the present study seeks to address this gap by examining whether quiet quitting is associated with nurses’ perceptions of the quality and safety of care.

## 2. Materials and Methods

### 2.1. Study Design

A cross-sectional study was conducted in Greece and data were collected using an online survey from October to December 2025. Google Forms was utilized for the questionnaire to be administered via nurses’ social media groups such as Facebook, Instagram and LinkedIn. Nurse’s networks derived from the nursing professional and institutional association networks. Specifically, the survey link was posted in nursing-related groups and pages and was also shared through professional association networks, enabling broad circulation among registered nurses. Participation was voluntary, and eligibility was limited to practicing nurses currently working in healthcare settings in Greece. No sampling frame was available, and no direct invitation list was used. Consequently, a non-probability convenience sampling approach was employed, whereby participants self-selected into the study upon exposure to the survey link. In addition, a snowballing element was present, as participants were encouraged to share the survey within their professional networks, further expanding its reach. Given the open online dissemination strategy, it was not possible to determine the total number of nurses who received or viewed the survey invitation; therefore, a response rate could not be calculated. While this approach enabled the recruitment of nurses from diverse geographical regions and healthcare settings throughout Greece, the use of convenience and snowball sampling may limit the representativeness of the sample and should be considered when interpreting the findings. Participants were eligible if they were registered nurses currently employed in hospitals in Greece. Recruitment relied on self-selection, whereby individuals who encountered the survey invitation and met the eligibility criteria could voluntarily participate. A snowball sampling component was also incorporated, as participants were encouraged to forward the survey link to colleagues within their professional networks. Consequently, the final sample consisted of nurses who chose to participate after exposure to the survey invitation. No hospitals or healthcare organizations were selected or recruited as study sites. Instead, the survey was disseminated nationally through online professional networks, allowing participation from nurses working across a variety of public and private hospitals. Because recruitment was not tied to specific institutions, the exact distribution of participating healthcare organizations could not be determined.

### 2.2. Eligibility Criteria and Sample Size Calculation

Eligibility criteria in this study were the following: (a) clinical nurses who were working in hospital settings; (b) subordinates—not managers or seniors of other nurses—who provided direct patient care for more than one year in their position and (c) nurses willing to participate in the study by providing informed consent. Eligible participants were registered nurses currently employed in hospital settings in Greece and involved in the provision of direct patient care. To ensure that participants had sufficient exposure to the clinical work environment and organizational conditions relevant to the study variables, only nurses with at least one year of work experience in their current position were included. This criterion was applied to ensure that participants had adequate familiarity with their workplace culture, patient safety practices, quality of care procedures, and working conditions before evaluating issues related to quiet quitting. The study focused on frontline nursing staff; therefore, nurses occupying managerial, supervisory, or executive positions (e.g., nurse managers, head nurses, directors of nursing services, and other senior administrative personnel) were excluded. This decision was made because managerial personnel have different roles, responsibilities, and organizational perspectives that may influence both workplace attitudes and perceptions of healthcare quality and safety. By restricting participation to subordinate nurses providing direct patient care, a more homogeneous study population was obtained. Additional inclusion criteria were the ability to read and understand Greek, access to the online questionnaire, and willingness to participate in the study. Prior to accessing the survey questions, all participants were provided with information regarding the study objectives, voluntary nature of participation, confidentiality of responses, and data protection procedures. Electronic informed consent was obtained from all participants before survey completion. Nurses who were not actively employed in hospital clinical practice at the time of data collection, including retired nurses, nursing students, interns, administrative nursing personnel, and nurses working exclusively in managerial positions, were deemed ineligible and excluded from participation.

The study complied with the Strengthening the Reporting of Observational studies in Epidemiology (STROBE) guideline [24].

We used the G*Power v.3.1.9.2 software to calculate our sample size. Our models included one predictor (quiet quitting) and five confounders. Therefore, based on an expected effect size of 0.03, a statistical power of 95%, and an alpha level of 0.05, the minimum required sample size was estimated to be 436 nurses.

### 2.3. Measurements

The demographic characteristics assessed were gender, age, years of professional experience, shift work status, and employment in an understaffed department.

Quiet quitting among nurses was measured using the 9-item Quiet Quitting Scale (QQS) [25]. Responses were rated at a 5-point Likert-type scale ranging from one (very strongly disagree/never) to five (strongly agree/always). It is composed of three factors, i.e., “detachment” (four items), “lack of initiative” (three items) and “lack of motivation” (two items). Factor scores were computed as the mean value of the item answers (range: 1–5). Higher levels of quiet quitting are associated with higher scores. The validated Greek version of the QQS was employed in this study [10]. Internal consistency was satisfactory, with a Cronbach’s alpha of 0.821 for the total scale, and 0.756 for detachment, 0.734 for lack of initiative, and 0.820 for lack of motivation.

Work environment among nurses was measured using the 5-item short form of the Practice Environment Scale of the Nursing Work Index (PES-5) [26]. The 5 items are: (1) Administration that listens and responds to nurse concerns, (2) A supervisor who is a good manager and leader, (3) Good teamwork between nurses and physicians, (4) Enough staff to get work done, (5) A clear philosophy of nursing that pervades the patient care environment. Responses were rated at a 4-point Likert-type scale ranging from one (strongly disagree) to four (strongly agree). Responses to the five items were summed and divided by five to calculate a composite mean score for each participant. The resulting score ranged from one to four, with higher values indicate better realization of a supportive work environment. The validated version of the PES-5 in Greek was used [27]. In our study, Cronbach’s alpha for the PES-5 was 0.735.

Perceived quality of care was measured with the question, “How do you evaluate the quality of care in your unit?”, with responses recorded on a four-point Likert scale ranging from poor to fair, good, and excellent. This single-item measure represents a reliable approach to assessing the quality of nursing care delivery and is widely used in international research [28].

A single-item measure was used to assess patient safety, asking respondents to rate patient safety in their unit on a five-point Likert scale ranging from very poor to excellent. For analysis’s purposes, both items were dichotomized (0 = very poor/poor/fair, 1 = good/excellent) as described in the literature [29]. Higher values reflect higher perceived quality of care and patient safety ratings.

### 2.4. Ethical Issues

Our research was performed in compliance with the Declaration of Helsinki principles [30]. The Ethics Committee of the Faculty of Nursing, National and Kapodistrian University of Athens approved our research protocol (approval number: 08, 23 September 2025). Participation was anonymous and voluntary. All participants were informed about the aim and the design of study and their consent was obtained by the investigators.

### 2.5. Statistical Analysis

Descriptive statistics for continuous variables are presented as means, standard deviations (SDs), medians, and interquartile ranges, whereas categorical variables are summarized using frequencies and percentages. The normality of continuous variables was assessed using the Kolmogorov–Smirnov test and Q–Q plots. Quiet quitting was specified as the predictor variable, while perceived quality of care and perceived patient safety were treated as outcome variables. Demographic and work-related factors were included as potential confounders.

Associations between quiet quitting and perceived quality of care and patient safety were examined using univariable and multivariable logistic regression analyses. Crude associations were initially explored through unadjusted univariable models, followed by multivariable logistic regression models to evaluate the independent effect of quiet quitting after adjustment for confounders. We found a very high correlation between age and years of work experience (r = 0.912, *p* < 0.001) which indicated a multicollinearity issue. Thus, only one of these two variables was included in the final multivariable models (years of work experience instead of age). Results are presented as unadjusted and adjusted odds ratios (ORs) with 95% confidence intervals (CIs), and *p*-values. Model fit was assessed using standard methods for logistic regression. There was no missing data. *p*-values < 0.05 were regarded as statistically significant. Statistical analyses were conducted using the IBM SPSS 28.0 (IBM Corp. Released 2021. IBM SPSS Statistics for Windows, Version 28.0. Armonk, NY, USA: IBM Corp.)

## 3. Results

### 3.1. Demographic Characteristics

Our sample included 492 nurses. Demographic characteristics of the sample are shown in Table 1. More than eight out of ten (82.1%, n = 404) of the participants were females, while 17.9% (n = 88) were males. Nurses’ mean age was 42.98 years old (SD = 18.27), and mean work experience was 9.79 years (SD = 9.95). Most of nurses worked in shifts (81.7%, n = 402), and in understaffed departments (85.2%, n = 419).

### 3.2. Study Scales

Table 2 depicts descriptive statistics for the Quiet Quitting Scale. The overall quiet quitting rating was low to moderate (mean = 2.18, SD = 0.65). Lack of motivation (mean = 2.77, SD = 1.00) had the highest mean score followed by lack of initiative (mean = 2.12, SD = 0.84) and detachment (mean = 1.93, SD = 0.73), indicating that motivational withdrawal was more pronounced than behavioral or emotional disengagement in this sample population. The mean score for the PES-5 was 2.44 (SD = 0.53) indicating a moderate realization of a supportive work environment.

Descriptive statistics for the three factors and nine items of the QQS are presented in Table 3. Mean score on factor “lack of motivation” (2.9) was higher than the other two factors (i.e., “lack of initiative” = 2.1, “detachment” = 1.9).

### 3.3. Quality of Care and Patient Safety

More than half of nurses (52.0%, n = 256) reported good quality of care in their unit, whereas 23.6% (n = 116) reported fair quality, 19.7% (n = 97) excellent quality, and 4.7% (n = 23) poor quality of care. In terms of patient safety, 33.1% (n = 163) of nurses rated it as good, 28.5% (n = 140) as very good, 20.7% (n = 102) as fair, 11.2% (n = 55) as excellent, and 6.5% (n = 32) as poor.

### 3.4. Outcome: Perceived Quality of Care

The unadjusted and adjusted effects of quiet quitting and nursing work environment on respondents’ perception of quality care are shown in Table 4, based on univariable and multivariable logistic regressions. Results from the univariable model showed that decreases in quiet quitting scores were significantly associated with higher odds of reporting good or excellent perceived quality of care. This association persisted in the multivariable model adjusted for demographic variables, as lower quiet quitting remained predictive of increased odds for good or excellent perceived quality of care (adjusted OR = 0.429; 95% CI: 0.310–0.593; *p* < 0.001). These results suggest that quiet quitting was a significant predictor of perceived care quality which remains significant after adjusting for demographic and workplace characteristics.

In the univariable model, higher work environment scores were significantly associated with greater odds of reporting perceived quality of care as good or excellent. Similarly, in the multivariable model higher PES-5 scores were linked to more than nine-fold increased odds of reporting good or excellent perceived quality of care (adjusted OR = 9.155; 95% CI: 5.374–15.596; *p* < 0.001). These results suggest that a more favorable nursing practice environment is an important and independent predictor of better perceived quality of care, irrespective of the key demographic and work environment characteristics that were considered in this study.

### 3.5. Outcome: Perceived Patient Safety

The unadjusted and adjusted effects of quiet quitting and nursing work environment on respondents’ perception of patient safety are presented in Table 5, based on univariable and multivariable logistic regression analyses. In the univariable model, lower quiet quitting scores were significantly associated with increased odds of reporting perceived patient safety as good or excellent (unadjusted OR = 0.423; 95% CI: 0.281–0.553; *p* < 0.001). This association remained statistically significant in the multivariable model after adjusting for gender, years of work experience, working in shifts, and working at an understaffed department. Specifically, lower quiet quitting continued to predict higher odds of good or excellent perceived patient safety (adjusted OR = 0.394; 95% CI: 0.281–0.553; *p* < 0.001). These findings indicate that quiet quitting was a significant predictor of perceived patient safety, and this association persists even after controlling for confounders. Results from the univariable model indicated a significant positive association between PES-5 scores and the odds of perceiving patient safety as good or excellent. This association continued to be significant after controlling for gender, years of work experience, working in shifts, and working in an understaffed department in the multivariable model. There was an over twenty-fold increase in the odds of reporting good or excellent perceived patient safety for high PES-5 scores (adjusted OR = 22.190; 95% CI: 11.533–42.693; *p* < 0.001). These findings suggest that a better practice environment for nurses is a robust and independent predictor of perceived patient safety after adjusting for important demographic and workplace variables. 

**Figure 1:**
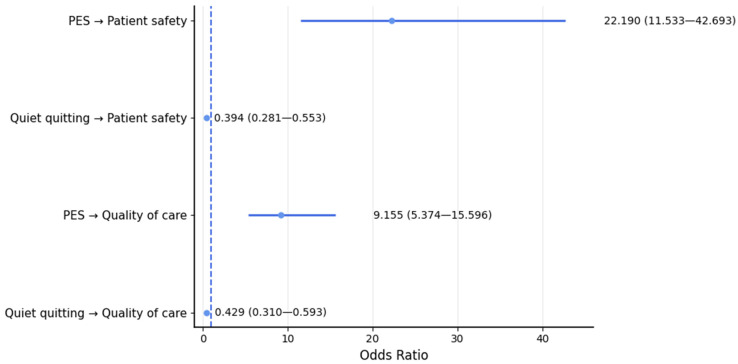
Forest plot for the association between quiet quitting, Practice Environment Scale (PES), patient safety, and quality of care.

Figure 1 shows the forest plot for the association between quiet quitting, Practice Environment Scale, patient safety, and quality of care.

## 4. Discussion

This study identified a moderate degree of quiet quitting among nurses, chiefly characterized by low motivation, a non-supportive work environment, and a significant association of quiet quitting and the nursing work environment with the quality and safety of the care delivered. Regarding the work environment and the quality and safety of care, our findings are consistent with those reported in the literature [5,31]. With respect to our findings on quiet quitting, the present study is the first to address this issue in the literature.

An interesting finding of the present study is that among the three dimensions of quiet quitting, “lack of motivation” demonstrated the highest mean score (2.9), compared with “lack of initiative” (2.1) and “detachment” (1.9). This pattern suggests that quiet quitting among nurses may primarily manifest as a gradual decline in intrinsic motivation rather than as overt withdrawal behaviors or complete psychological disengagement. In other words, nurses may continue to perform their required duties and maintain a basic level of professional functioning, while experiencing reduced enthusiasm, energy, and willingness to go beyond formal role expectations. This interpretation aligns with the demanding nature of nursing work, where professional norms and ethical responsibility toward patient care may prevent full detachment, even in the presence of reduced motivation. Furthermore, the comparatively lower levels of “detachment” may indicate that, despite adverse work conditions, nurses retain a degree of emotional and professional connection to their work and patients. However, the elevated “lack of motivation” highlights a potential early stage of disengagement, which, if unaddressed, could progress toward more pronounced forms of withdrawal, including reduced initiative and eventual detachment. These findings underscore the importance of organizational strategies aimed at enhancing motivation, such as supportive leadership, adequate staffing, and recognition systems, as a means of preventing the escalation of quiet quitting behaviors and protecting care quality and patient safety.

The moderate score on the lack of initiative subscale and the high score on the lack of motivation subscale highlight nurses’ passive stance. During and following the COVID-19 pandemic, many nurses left the profession. However, those who remained did not necessarily do so because they were satisfied with their working conditions; rather, they may have stayed because of concerns about securing alternative employment and the need to fulfill family obligations. To preserve their work–life balance and prioritize their well-being, some nurses may consequently engage in quiet quitting [32]. However, nursing requires sustained vigilance and continuous professional development. Nurses are expected to remain up to date with the latest scientific evidence and integrate it into daily clinical practice, including relevant clinical guidelines; use emerging technologies effectively, including artificial intelligence applications; and have opportunities to implement innovative practices aimed at improving the quality of nursing care [33,34,35]. The support nurses receive from their supervisors regarding innovation, such as idea generation, idea search, idea communication, implementation-initiation activities, involving others, and overcoming obstacles, contributes both to improved innovation outputs and to a reduced likelihood of quiet quitting [36]. When the nursing work environment is characterized by appreciation, support, and professional development opportunities, nurses tend to be more motivated, exhibit a lower intention to leave their positions, and are less likely to develop burnout, while their physical health and job performance also improve [37,38,39]. By enacting leadership behaviors that promote and secure autonomy, competence, relatedness, supportive relationships, and organizational support, nurse managers can motivate nurses, strengthen their work engagement, and enhance their job performance [40].

Among the components of the nursing practice environment, staffing adequacy remains one of the most persistent and challenging issues for healthcare organizations [41]. Insufficient nurse staffing can affect care outcomes through multiple pathways. At the patient level, inadequate staffing has been associated with increased mortality, higher readmission rates, longer hospital stays, and a greater likelihood of nurse-sensitive adverse events [17,42]. At the workforce level, chronic understaffing intensifies nurses’ workload and may contribute to burnout, job dissatisfaction, psychological strain, fatigue, and stronger intentions to leave the job or profession [43,44]. These workforce-related consequences can further weaken nurses’ capacity to provide timely, comprehensive, and safe care, thereby indirectly undermining both the quality of nursing care and patient safety [45,46,47].

Supportive leadership that actively listens to and addresses nurses’ concerns and challenges, fosters effective communication and interdisciplinary collaboration, and provides a clear framework for nursing practice appears to be a key prerequisite for the delivery of high-quality and safe patient care [48]. These leadership attributes were shown to be particularly important during the COVID-19 pandemic and the challenging conditions it created, as they contributed to enhancing nurses’ resilience [49], which in turn was associated with better quality of care [50]. During the pandemic, nurses often experienced a sense of powerlessness in coping with the exceptionally high demands of their work. Nevertheless, support from colleagues and supervisors appeared to play a critical role in enabling them to manage these challenges more effectively, improve their mental well-being, and lessen the likelihood of turnover intention [51].

Promoting a culture of safety that supports both error prevention and effective error management is another key responsibility of nursing leadership. A mature safety culture depends on leadership that creates conditions for learning, transparency, and shared responsibility. In this context, leaders should promote the systematic use of errors as a source of organizational learning, support fair and blame-free responses to adverse events, facilitate open communication across professional groups, and ensure that staff are actively engaged in decisions affecting clinical practice and patient safety [48]. Conversely, when nurse managers engage in toxic behaviors, such as psychological manipulation (gaslighting), the quality and safety of care are adversely affected [52].

This study has several limitations that should be carefully considered when interpreting the findings. First, its cross-sectional design constitutes an important methodological constraint, as it precludes any inference regarding causal relationships or the temporal sequencing of the observed associations. Specifically, while significant associations were identified between quiet quitting, the nursing work environment, and outcomes related to quality of care and patient safety, it cannot be determined whether unfavorable work environments and quiet quitting behaviors lead to poorer care and safety outcomes, or whether challenges in delivering safe and high-quality care contribute to disengagement and quiet quitting among nurses. Furthermore, the possibility of bidirectional or reciprocal associations cannot be ruled out. Longitudinal or prospective study designs are therefore necessary to clarify the directionality, stability, and potential causal mechanisms underlying these associations over time. Second, the evaluation of care quality and patient safety relied exclusively on self-reported data, without the inclusion of objective administrative indicators or clinical information extracted from patient records. Therefore, the findings may be influenced by respondents’ subjective perceptions, as well as by potential reporting bias. In addition, the use of a convenience sample of nurses to examine the associations among the nursing work environment, quiet quitting, quality of care, and patient safety limits the external validity of the study and constrains the extent to which the findings can be generalized to the broader nursing workforce in Greece and other healthcare contexts. Future studies should adopt random, stratified, and more representative sampling strategies to strengthen the methodological rigor, robustness, and generalizability of the evidence. As this study appears, to the best of our knowledge, to be the first to investigate the association between quiet quitting and the quality and safety of care, additional research in diverse healthcare systems, organizational settings, and cultural contexts is needed to validate, further clarify, and expand upon the present findings. Furthermore, the strong association observed between the nursing work environment (PES-5) and patient safety outcomes (OR = 22.19) should be interpreted with caution. As all key study variables (i.e., nursing work environment, quiet quitting, quality of care, and patient safety) were measured using self-reported data from the same respondents and collected at a single point in time, the findings may be affected by common method (same-source) bias. This type of bias can inflate the observed associations due to shared variance attributable to individual response tendencies, such as mood, cognitive framing, or social desirability, rather than reflecting true underlying associations. Consequently, the magnitude of the reported associations may be overestimated. Future research should seek to mitigate this limitation by employing multi-source and multi-method approaches, such as combining nurse-reported measures with objective patient safety indicators, supervisor assessments, or administrative data. In addition, longitudinal and multi-level study designs would help disentangle individual perceptions from organizational effects and provide a more accurate estimation of the associations among the nursing work environment, quiet quitting, and patient outcomes. In addition, data were collected through recruitment via social media platforms, which precluded the calculation of a response rate, as the total number of individuals who were exposed to the study invitation could not be determined. This limitation reduces the transparency of sample participation and makes it difficult to assess potential non-response bias. Moreover, social media-based sampling may introduce self-selection bias, as participation is more likely among nurses who are more digitally engaged, motivated to express their views, or who have stronger opinions or experiences related to their work environment and professional engagement. Consequently, the sample may not fully reflect the characteristics and experiences of the broader nursing population, which may affect the representativeness and generalizability of the findings. Future studies should consider alternative or complementary recruitment strategies that allow for better estimation of response rates and more controlled sampling procedures. Moreover, the use of convenience sampling through an online survey introduces a risk of selection bias. Participation was voluntary and unrestricted, which resulted in a non-random sample of nurses. Individuals who chose to respond may differ systematically from those who did not participate, for example in terms of their level of work engagement, job satisfaction, or experiences with the work environment and patient safety. As a result, certain perspectives may be overrepresented, while others may be underrepresented or absent. This potential selection bias limits the internal validity of the findings and further constrains their generalizability to the wider nursing population. Future studies should aim to use probability-based sampling methods and more structured recruitment procedures to reduce selection bias and enhance representativeness. In relation to the cross-sectional design, an important limitation concerns the inability to establish the temporal ordering of the key variables examined in this study. Although associations were identified between quiet quitting, the nursing work environment, quality of care, and patient safety, it is not possible to determine whether the work environment influences quiet quitting behaviors, which in turn affect care quality and safety, or whether poorer quality and safety conditions contribute to disengagement and the emergence of quiet quitting. Similarly, the associations among these variables may be dynamic and reciprocal over time, rather than unidirectional. The lack of temporal sequencing limits the ability to test potential causal pathways. Longitudinal, prospective, or panel study designs are needed to clarify the directionality, temporal stability, and causal structure of these associations and to more rigorously examine underlying mechanisms. Another important limitation is the absence of several potentially relevant individual- and work-related variables, such as clinical department, workload, staffing levels, and compensation. These factors have been shown to influence both quiet quitting among nurses and perceptions of care quality and patient safety. The omission of such variables raises the possibility of residual confounding, as the observed associations between quiet quitting, the nursing work environment, and study outcomes may be partially explained by unmeasured factors. For example, nurses working in high-intensity departments or under conditions of increased workload or shift burden may be more likely to report quiet quitting as well as lower perceived quality and safety of care. Future studies should incorporate a broader set of organizational, occupational, and demographic variables and apply multivariable or stratified analytical approaches to better account for confounding influences and to provide a more nuanced understanding of these associations. Finally, quality of care and patient safety were assessed using single-item measures, which may be more vulnerable to measurement error compared to multi-item, validated scales. Single-item indicators may not fully capture the multidimensional nature of these complex constructs and may be less reliable, potentially leading to reduced precision and stability of the estimates. As a result, the observed associations may be attenuated or, in some cases, inflated due to random measurement error. Future research should consider the use of comprehensive, psychometrically validated multi-item instruments and, where possible, the inclusion of objective quality and safety indicators to enhance the reliability and validity of outcome measurement.

## 5. Conclusions

The quality and safety of patient care have remained central priorities for healthcare organizations worldwide for decades. The findings of the present study indicate that fostering a healthy nursing work environment, characterized by adequate staffing, organizational support, and recognition from nursing leadership, may contribute substantially to improving the quality and safety of nursing care. Conversely, demanding working conditions and the absence of a supportive work environment may promote quiet quitting among nurses, which, in turn, may compromise care quality and patient safety. Improving the nursing work environment is therefore fundamental to strengthening the quality and safety of nursing care. These findings provide valuable insights for policymakers and healthcare managers involved in the organization and delivery of healthcare services.

## Figures and Tables

**Table 1:** Demographic characteristics of the sample (n = 492).

Characteristics	N	%
Sex		
Males	88	17.9
Females	404	82.1
Age, mean, standard deviation	42.98	18.27
Work experience, mean, standard deviation	9.79	9.95
Shifts		
No	90	18.3
Yes	402	81.7
Understaffed departments		
No	73	14.8
Yes	419	85.2

**Table 2:** Descriptive statistics for the study scales (n = 492).

Scale	Mean	Standard Deviation	Median	Interquartile Range
Quiet Quitting Scale	2.18	0.65	2.11	0.86
Detachment	1.93	0.73	1.88	0.75
Lack of initiative	2.12	0.84	2.00	1.33
Lack of motivation	2.77	1.00	2.50	1.50
Practice Environment Scale	2.44	0.53	2.40	0.60

**Table 3:** Descriptive statistics for the three factors and nine items of the Quiet Quitting Scale.

			Answers	
Items	Mean	Standard Deviation	Median	Interquartile Range
Score on factor “detachment”	1.9	0.7	1.9	0.8
I do the basic or minimum amount of work without going above and beyond.	2.0	1.0	2.0	1.0
If a colleague can do some of my work, then I let him/her do it.	2.4	1.1	2.0	1.0
I take as many breaks as I can.	1.8	0.9	2.0	1.0
How often do you pretend to be working in order to avoid another task?	1.6	0.8	1.0	1.0
Score on factor “lack of initiative”	2.1	0.8	2.0	1.3
I don’t express opinions and ideas about my work because I am afraid that the manager assigns me more tasks.	1.8	0.9	2.0	1.0
I don’t express opinions and ideas about my work because I think that work conditions are not going to change.	2.4	1.3	2.0	2.0
I do not take initiative at my work	2.2	1.0	2.0	1.0
Score on factor “lack of motivation”	2.8	1.0	2.5	1.5
I do not find motives in my job.	2.6	1.1	2.0	1.0
I do not feel inspired when I work.	2.9	1.1	3.0	2.0

Higher values indicate higher levels of quiet quitting.

**Table 4:** Logistic regression models with perceived quality of care as the dependent variable (n = 492).

Independent Variable	Univariable Model			Multivariable Model ^a^		
	Unadjusted OR	95% CI for OR	*p*-Value	Adjusted OR	95% CI for OR	*p*-Value
Quiet quitting	0.429	0.313 to 0.588	<0.001	0.429	0.310 to 0.593	<0.001
Practice Environment Scale	7.686	4.681 to 12.620	<0.001	9.155	5.374 to 15.596	<0.001

^a^ Multivariable model is adjusted for gender, years of work experience, working in shifts and working at an understaffed department. OR: Odds Ratio; CI: Confidence Interval.

**Table 5:** Logistic regression models with perceived patient safety as the dependent variable (n = 492).

Independent Variable	Univariable Model			Multivariable Model ^a^		
	Unadjusted OR	95% CI for OR	*p*-Value	Adjusted OR	95% CI for OR	*p*-Value
Quiet quitting	0.423	0.308 to 0.581	<0.001	0.394	0.281 to 0.553	<0.001
Practice Environment Scale	21.151	11.301 to 39.585	<0.001	22.190	11.533 to 42.693	<0.001

^a^ Multivariable model is adjusted for gender, years of work experience, working in shifts and working at an understaffed department. OR: Odds Ratio; CI: Confidence Interval.

## Data Availability

The data used in this study are openly available in Figshare at: https://figshare.com/articles/dataset/gaslighting/31566319?file=62512651 (accessed on 1 June 2026).

## References

[1] Laskowski-Jones L., Castner J. (2022). The Great Resignation, Newly Licensed Nurse Transition Shock, and Emergency Nursing. J. Emerg. Nurs..

[2] Guttormson J.L., Calkins K., McAndrew N., Fitzgerald J., Losurdo H., Loonsfoot D. (2022). Critical Care Nurse Burnout, Moral Distress, and Mental Health During the COVID-19 Pandemic: A United States Survey. Heart Lung.

[3] Getie A., Ayenew T., Amlak B.T., Gedfew M., Edmealem A., Kebede W.M. (2025). Global Prevalence and Contributing Factors of Nurse Burnout: An Umbrella Review of Systematic Review and Meta-Analysis. BMC Nurs..

[4] Lasater K.B. (2024). Addressing the Nurse Retention Crisis—Leveraging Policies Supported by Evidence. JAMA Netw. Open.

[5] Lake E.T., Sanders J., Duan R., Riman K.A., Schoenauer K.M., Chen Y. (2019). A Meta-Analysis of the Associations Between the Nurse Work Environment in Hospitals and 4 Sets of Outcomes. Med. Care.

[6] NCSBN NCSBN Research Projects Significant Nursing Workforce Shortages and Crisis. https://www.ncsbn.org/news/ncsbn-research-projects-significant-nursing-workforce-shortages-and-crisis.

[7] Gün İ., Kutun F.Ç., Söyük S. (2025). Mediating Effect of Turnover Intention on the Relationship Between Job Burnout and Quiet Quitting in Nurses. J. Adv. Nurs..

[8] Harter J. Is Quiet Quitting Real?. https://www.gallup.com/workplace/398306/quiet-quitting-real.aspx.

[9] Rinaldi S., Pomarolli E. (2025). Quiet Quitting Among Nurses: A Case Study in a Northern Italian Hospital. Nurs. Rep..

[10] Galanis P., Katsiroumpa A., Vraka I., Siskou O., Konstantakopoulou O., Katsoulas T., Moisoglou I., Gallos P., Kaitelidou D. (2024). Nurses Quietly Quit Their Job More Often than Other Healthcare Workers: An Alarming Issue for Healthcare Services. Int. Nurs. Rev..

[11] Hungerford C., Jackson D., Cleary M. (2025). Quiet Quitting, Resenteeism and Other Forms of Disengagement: What Are the Answers for Nurses?. J. Adv. Nurs..

[12] Zuzelo P.R. (2023). Discouraging Quiet Quitting: Potential Strategies for Nurses. Holist. Nurs. Pract..

[13] Galanis P., Moisoglou I., Katsiroumpa A., Gallos P., Kalogeropoulou M., Meimeti E., Vraka I. (2025). Workload Increases Nurses’ Quiet Quitting, Turnover Intention, and Job Burnout: Evidence from Greece. AIMS Public Health.

[14] Moisoglou I., Katsiroumpa A., Katsapi A., Konstantakopoulou O., Galanis P. (2025). Poor Nurses’ Work Environment Increases Quiet Quitting and Reduces Work Engagement: A Cross-Sectional Study in Greece. Nurs. Rep..

[15] Moisoglou I., Katsiroumpa A., Papathanasiou I.V., Konstantakopoulou O., Katharaki M., Malliarou M., Tsaras K., Prasini I., Rekleiti M., Galanis P. (2025). Engaging Leadership Reduces Quiet Quitting and Improves Work Engagement: Evidence from Nurses in Greece. Nurs. Rep..

[16] Kramer M. (1990). The Management Hospitals: Excellence Revisited. JONA J. Nurs. Adm..

[17] Morioka N., Moriwaki M., Miyawaki A., Saville C., Fushimi K., Griffiths P. (2026). Hospital Nurse Understaffing and Patient Mortality, Readmission, and Length of Stay. JAMA Netw. Open.

[18] Kutney-Lee A., Germack H., Hatfield L., Kelly S., Maguire P., Dierkes A., Del Guidice M., Aiken L.H. (2016). Nurse Engagement in Shared Governance and Patient and Nurse Outcomes. JONA J. Nurs. Adm..

[19] Boamah S. (2018). Linking Nurses’ Clinical Leadership to Patient Care Quality: The Role of Transformational Leadership and Workplace Empowerment. Can. J. Nurs. Res..

[20] Asif M., Jameel A., Hussain A., Hwang J., Sahito N. (2019). Linking Transformational Leadership with Nurse-Assessed Adverse Patient Outcomes and the Quality of Care: Assessing the Role of Job Satisfaction and Structural Empowerment. Int. J. Environ. Res. Public Health.

[21] Kang X.L., Brom H.M., Lasater K.B., McHugh M.D. (2020). The Association of Nurse-Physician Teamwork and Mortality in Surgical Patients. West J. Nurs. Res..

[22] Boev C., Xia Y. (2015). Nurse-Physician Collaboration and Hospital-Acquired Infections in Critical Care. Crit. Care Nurse.

[23] Halm M. (2019). The Influence of Appropriate Staffing and Healthy Work Environments on Patient and Nurse Outcomes. Am. J. Crit. Care.

[24] von Elm E., Altman D.G., Egger M., Pocock S.J., Gøtzsche P.C., Vandenbroucke J.P. (2008). The Strengthening the Reporting of Observational Studies in Epidemiology (STROBE) Statement: Guidelines for Reporting Observational Studies. J. Clin. Epidemiol..

[25] Galanis P., Katsiroumpa A., Vraka I., Siskou O., Konstantakopoulou O., Moisoglou I., Gallos P., Kaitelidou D., Galanis P., Katsiroumpa A. (2023). The Quiet Quitting Scale: Development and Initial Validation. AIMS Public Health.

[26] Lake E.T., Gil J., Moronski L., Mchugh M.D., Aiken L.H., Lasater K.B. (2024). Validation of a Short Form of the Practice Environment Scale of the Nursing Work Index: The PES-5. Res. Nurs. Health.

[27] Katsiroumpa A., Moisoglou I., Konstantakopoulou O., Kalogeropoulou M., Gallos P., Tsiachri M., Galanis P. (2024). Practice Environment Scale of the Nursing Work Index (5 Items Version): Translation and Validation in Greek. Int. J. Caring Sci..

[28] Muir K.J., Sloane D.M., Aiken L.H., Hovsepian V., McHugh M.D. (2023). The Association of the Emergency Department Work Environment on Patient Care and Nurse Job Outcomes. JACEP Open.

[29] Lake E.T., Hallowell S.G., Kutney-Lee A., Hatfield L.A., Del Guidice M., Boxer B.A., Ellis L.N., Verica L., Aiken L.H. (2016). Higher Quality of Care and Patient Safety Associated With Better NICU Work Environments. J. Nurs. Care Qual..

[30] World Medical Association World Medical Association (2013). Declaration of Helsinki: Ethical Principles for Medical Research Involving Human Subjects. JAMA.

[31] Wei H., Sewell K.A., Woody G., Rose M.A. (2018). The State of the Science of Nurse Work Environments in the United States: A Systematic Review. Int. J. Nurs. Sci..

[32] Serenko A. (2023). The Human Capital Management Perspective on Quiet Quitting: Recommendations for Employees, Managers, and National Policymakers. J. Knowl. Manag..

[33] Cacchione P.Z. (2020). Innovative Care Models across Settings: Providing Nursing Care to Older Adults. Geriatr. Nurs..

[34] Yang Y.T., Ricciardi R. (2026). Regulating AI in Nursing and Healthcare: Ensuring Safety, Equity, and Accessibility in the Era of Federal Innovation Policy. Policy Politics Nurs. Pract..

[35] Cato K.D., Sun C., Carter E.J., Liu J., Rivera R., Larson E. (2019). Linking to Improve Nursing Care and Knowledge: Evaluation of an Initiative to Provide Research Support to Clinical Nurses. JONA J. Nurs. Adm..

[36] Moisoglou I., Katsiroumpa A., Prasini I., Gallos P., Kalogeropoulou M., Galanis P. (2024). Innovation Support Reduces Quiet Quitting and Improves Innovative Behavior and Innovation Outputs among Nurses in Greece. Nurs. Rep..

[37] Gunawan N.P.I.N., Hariyati R.T.S., Gayatri D. (2019). Motivation as a Factor Affecting Nurse Performance in Regional General Hospitals: A Factors Analysis. Enferm. Clín..

[38] Dill J., Erickson R.J., Diefendorff J.M. (2016). Motivation in Caring Labor: Implications for the Well-Being and Employment Outcomes of Nurses. Soc. Sci. Med..

[39] Kurucová R., Čáp J., Bóriková I., Tomagová M., Kohanová D. (2025). Factors Contributing to Work Motivation of Nurses: Synthesis of Qualitative Studies. Contemp. Nurse.

[40] Alsadaan N., Salameh B., Reshia F.A.A.E., Alruwaili R.F., Alruwaili M., Awad Ali S.A., Alruwaili A.N., Hefnawy G.R., Alshammari M.S.S., Alrumayh A.G.R. (2023). Impact of Nurse Leaders Behaviors on Nursing Staff Performance: A Systematic Review of Literature. INQUIRY.

[41] Lasater K.B., Aiken L.H., Sloane D.M., French R., Martin B., Reneau K., Alexander M., McHugh M.D. (2021). Chronic Hospital Nurse Understaffing Meets COVID-19: An Observational Study. BMJ Qual. Saf..

[42] Juvé-Udina M.-E., Adamuz J., González-Samartino M., Tapia-Pérez M., Jiménez-Martínez E., Berbis-Morello C., Polushkina-Merchanskaya O., Zabalegui A., López-Jiménez M.-M. (2025). Association Between Nurse Staffing Coverage and Patient Outcomes in a Context of Prepandemic Structural Understaffing: A Patient-Unit-Level Analysis. J. Nurs. Manag..

[43] Shin S., Park J.-H., Bae S.-H. (2018). Nurse Staffing and Nurse Outcomes: A Systematic Review and Meta-Analysis. Nurs. Outlook.

[44] Bae S.-H. (2021). Intensive Care Nurse Staffing and Nurse Outcomes: A Systematic Review. Nurs. Crit. Care.

[45] Huang T.-L., Wong M.-K., Shyu Y.-I.L., Ho L.-H., Yeh J.-R., Teng C.-I. (2021). Reducing Turnover Intention to Improve Care Outcome: A Two-Wave Study. J. Adv. Nurs..

[46] Zabin L.M., Zaitoun R.S.A., Sweity E.M., de Tantillo L. (2023). The Relationship between Job Stress and Patient Safety Culture among Nurses: A Systematic Review. BMC Nurs..

[47] Li L.Z., Yang P., Singer S.J., Pfeffer J., Mathur M.B., Shanafelt T. (2024). Nurse Burnout and Patient Safety, Satisfaction, and Quality of Care: A Systematic Review and Meta-Analysis. JAMA Netw. Open.

[48] Murray M., Sundin D., Cope V. (2018). The Nexus of Nursing Leadership and a Culture of Safer Patient Care. J. Clin. Nurs..

[49] Sihvola S., Kvist T., Nurmeksela A. (2022). Nurse Leaders’ Resilience and Their Role in Supporting Nurses’ Resilience during the COVID-19 Pandemic: A Scoping Review. J. Nurs. Manag..

[50] Vaisi-Raygani A., Moradi M., Salari N., Fattahi Z., Hadadian F. (2025). Resilience and Nursing Care Quality in Emergency Departments: A 2024 Study in Kermanshah, Iran Hospitals. BMC Nurs..

[51] Miller J., Young B., Mccallum L., Rattray J., Ramsay P., Salisbury L., Scott T., Hull A., Cole S., Pollard B. (2024). “Like Fighting a Fire with a Water Pistol”: A Qualitative Study of the Work Experiences of Critical Care Nurses during the COVID-19 Pandemic. J. Adv. Nurs..

[52] Moisoglou I., Katsiroumpa A., Papathanasiou I.V., Konstantakopoulou O., Yfantis A., Katsapi A., Galanis P. (2026). Association Between Workplace Gaslighting and Perceived Quality of Care, Patient Safety and Quiet Quitting: A Cross-Sectional Study Among Nurses in Greece. Healthcare.

